# Benign Fibrous Histiocytoma of the Buccal Mucosa: Case Report and Literature Review

**DOI:** 10.1155/2010/306148

**Published:** 2010-06-17

**Authors:** Paraskevi Giovani, Anna Patrikidou, Aris Ntomouchtsis, Soultana Meditskou, Henri Thuau, Kostas Vahtsevanos

**Affiliations:** ^1^Department of Maxillofacial Surgical Oncology, “Theagenio” Cancer Hospital, 2 Al. Simeonidi Street, 540 07 Thessaloniki, Greece; ^2^Laboratory of Histology, Embryology and Anthropology, Medical School, Aristotle University of Thessaloniki, 54124 Thessaloniki, Greece; ^3^Craniofacial Unit, Chelsea and Westminster Hospital, London SW109NH, UK

## Abstract

Benign fibrous histiocytoma is an interesting and challenging entity even in its most usual, cutaneous presentation. Noncutaneous presentation is extremely limited, even more so for the mucosa of the head and neck area. We herein report such a case, describing the clinical characteristics of the lesion, complete diagnostic evaluation, management, and follow-up. Diagnostic histopathological challenges are specifically illustrated. A complete review of the relevant literature is also included.

## 1. Introduction

The benign fibrous histiocytoma (BFH) is a mesenchymal tumour that has been described as a benign neoplasm composed of fibroblasts and histiocytes arising in the cutaneous and noncutaneous soft tissues [1]. Because of the confusion over the natural history of fibrohistiocytic lesions, BFH was not identified as a separate clinical entity until the 1960s [2]. Cutaneous BFH commonly originates in sun-exposed skin. Non-cutaneous BFH represents approximately 1% of all benign FH lesions [3], and most frequently occurs in the soft tissues in the lower extremities (50%), less frequently in the upper extremities (20%), retroperitoneum (20%) [4].

The occurrence of this lesion in deep soft tissues of the head and neck has been rarely reported [2, 5]. An exception to this is BFH arising in the orbital tissues, representing the most frequent primary mesenchymal tumour of the orbit in adult patients [4, 6]. In the rest of the head and neck area, non-cutaneous BFH may arise in the buccal spaces, but cases of BFH of the tongue [7, 8], gingival or alveolar ridge [9, 10], mandible [11], maxilla [12], lower and upper lips, soft palate [13], and floor of the mouth have also been described [14]. Rare occurrences also include the nasal cavity and paranasal sinuses [15–17], larynx [18], trachea [19], temporomandibular joint [20], and submandibular and parotid glands [21–23]. It most often develops as a painless mass with specific symptoms caused by interference with the normal anatomy and physiology of the area in which they arise [2, 5]. Interestingly, primary BFH has recently also been reported in the mandibular bone [24–26]. 

In contrast to malignant fibrous histiocytoma (MFH), benign fibrous histiocytoma of the noncutaneous soft tissues of the head and neck has received little attention in the head and neck surgical literature.

BFH is seen mainly in adults and most frequently in young and middle aged women. Patients are usually under 50 years of age and have a history of sun exposure, trauma, or chronic infection, suggesting that BFH is a reactive disease [5, 9, 13].

This article describes a case of BFH of the buccal mucosa and discusses its clinical and pathological characteristics and management.

## 2. Case Report

**Figure 1:**
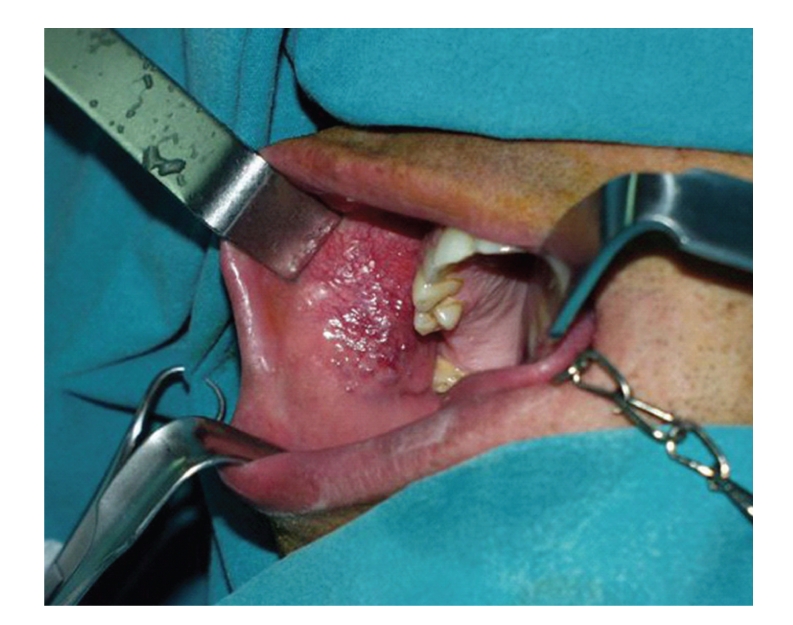
Buccal mucosa BFH: Preoperative view of the lesion.

A 36-year-old man was referred to the Department of Oral and Maxillofacial Surgery of “Theagenio” Cancer Hospital for an asymptomatic, slowly growing nodular lesion on the right buccal mucosa that had been present for approximately one year. His medical history was noncontributory. Examination revealed a well-circumscribed, moderately mobile, nontender fibroelastic lump of approximately 3 cm in diameter. The mucosal surface appeared smooth and moderately inflammatory, possibly due to local trauma (Figure 1).

**Figure 2:**
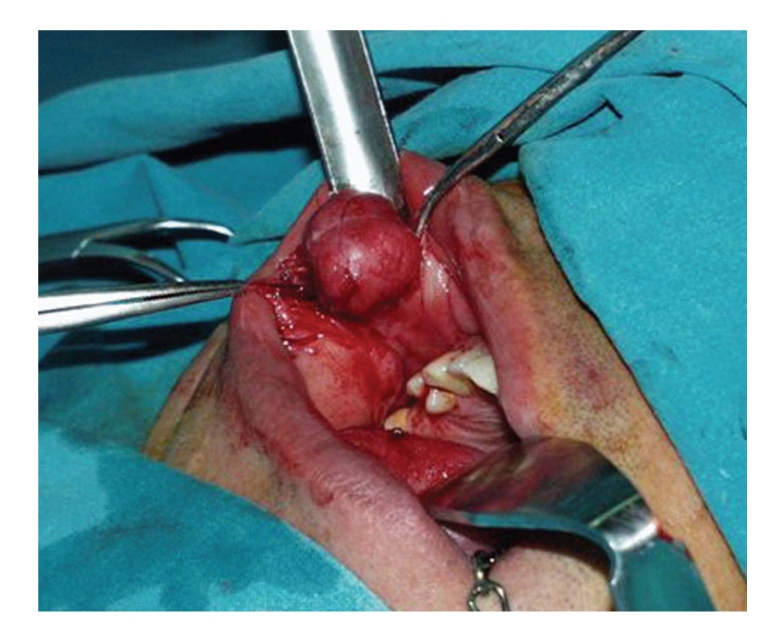
Buccal mucosa BFH: Intraoperative view of the lesion.

The patient underwent an intraoral excision of the mass under general anesthesia with primary closure. On surgical excision the mass was easily dissected from the surrounding tissues (Figure 2).

**Figure 3:**
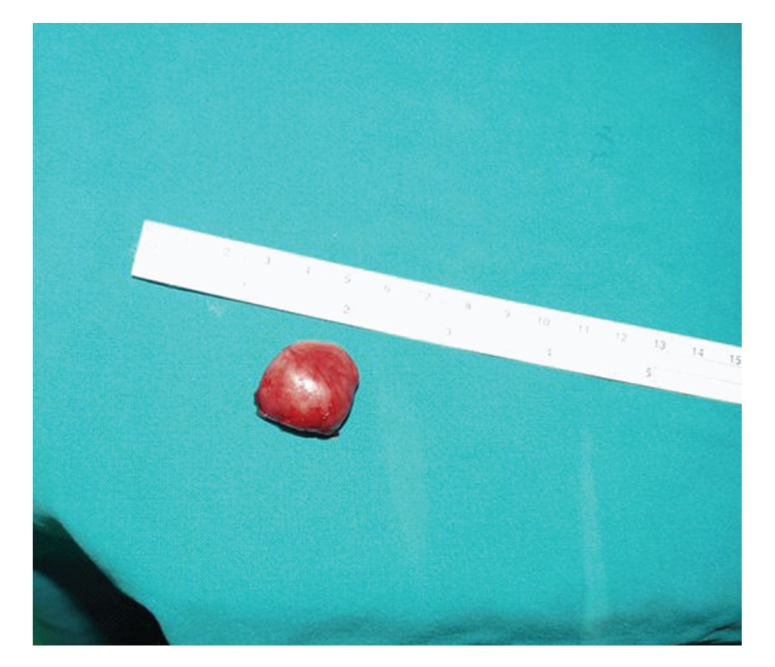
Buccal mucosa BFH: Surgical resection specimen.

**Figure 4:**
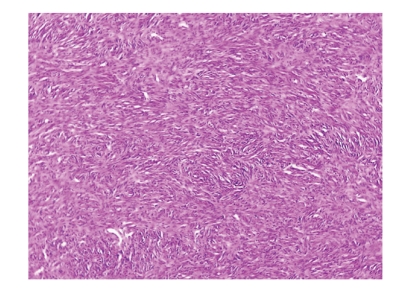
Spindle-shaped cells in a storiform pattern (H&E stain, ×40).

Histologic analysis of the specimen revealed a macroscopically smooth, well-circumscribed encapsulated lesion of 2.4 cm in greatest dimension. The mass was grossly round in appearance, with a white grey surface (Figure 3). Microscopically the tumour was composed of fibroblast-like spindle cells with fascicular and focally storiform arrangement (Figure 4). Plump, polygonal histiocytic cells were found among the spindle cells. There were no mitotic figures, cellular pleomorphism, multinuclear giant cells, nuclear atypia or necrosis. The stroma was collagenised and demonstrated a rich vascularity. There was a scattered inflammatory infiltrate predominantly composed of lymphocytes and plasma cells. 

**Figure 5:**
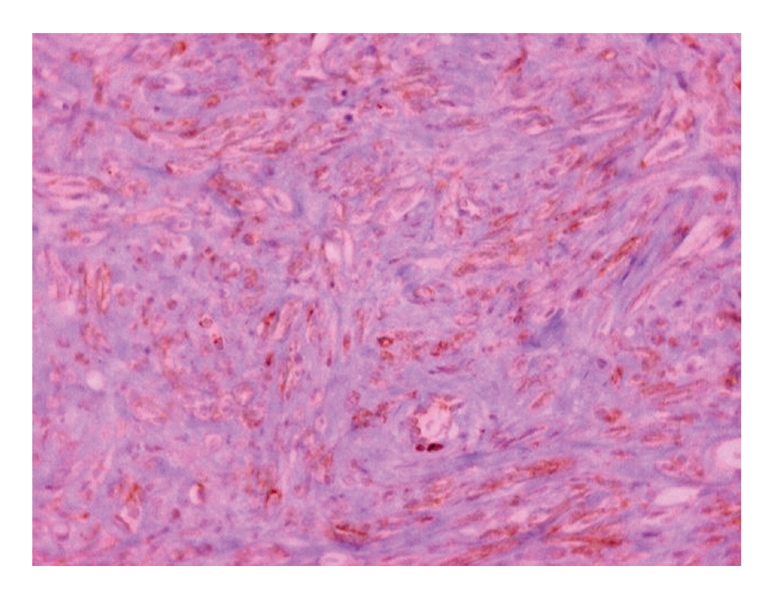
Spindle-shaped tumour cells were positive for CD34 (diaminobenzidine, ×400).

**Figure 6:**
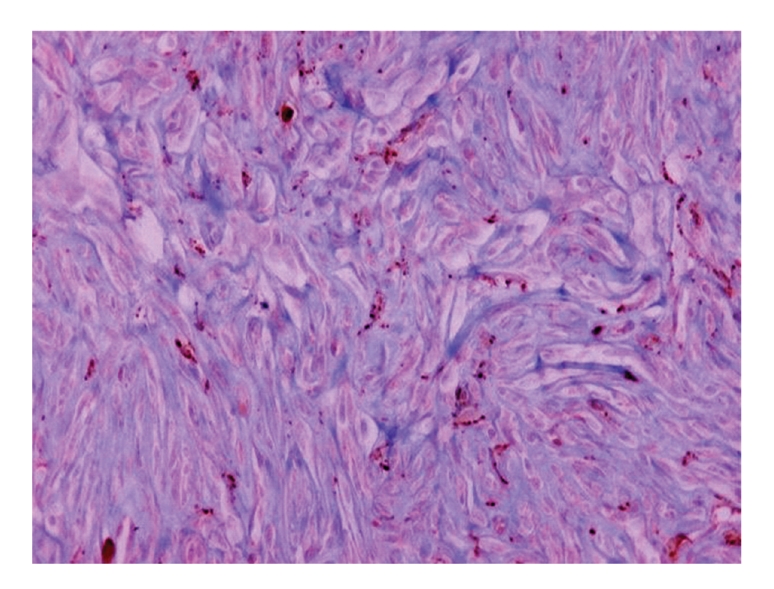
Histiocyte-like tumour cells were positive for CD68 (diaminobenzidine, ×400).

The tumour cells showed strong immunoreactivity for vimentin, weak and focal immunoreactivity for CD34 (Figure 5) and some tumour cells (histiocyte-like) were positive for CD68 (KP 1) (Figure 6). There was negativity for desmin, alpha smooth muscle actin (*α*-SMA), S-100 protein, Leu7, and CD117 (c-kit). 

The final diagnosis was BFH. The patient was free of loco-regional disease at 12-month follow-up.

## 3. Discussion

Pathologic analysis and diagnosis of this type of lesions is often challenging and usually based on a combination of light microscopy and immunohistochemistry [4].

Until recently the term fibrous histiocytoma (FH) referred to both benign and malignant neoplasms. The differential diagnosis between the two entities was often difficult [4]. The so-called malignant FH is not a definable entity but instead represents a wastebasket of undifferentiated pleomorphic sarcomas accounting for no more than 5% of adult soft tissue sarcomas. Although retained in the 2002 WHO classification of soft tissue tumours, it has long been recognised that these tumours have no relationship to true histocytes [27]. The benign fibrous histiocytoma also has a controversial diagnosis because of its uncertain histogenesis [2, 4, 11].

Due to the lack of specific markers for fibrohistiocytic lesions, the diagnosis of BFH is generally based on the absence of markers for cells of other lineages [4]. Immunohistochemical staining and ultrastructural examination of the tumours and cell lines derived from them has revealed features of myoblastic and histiocytic differentiation as evidence of mesenchymal origin [4]. Immunostaining for CD68 can be found in any tumour-containing lysosomal granules or phagolysosomes as in our case [4]. Factor XIII*α* has occasionally been reported for BFH [28].

In the present study, the diagnosis was confirmed using immunostaining for vimentin (+), CD68 (+), CD34 (+), S-100 (−), CD117 (−), Leu7 (−), desmin (−), and *α*-SMA (−).

Histopathologically, BFH typically shows a biphasic cell population of histiocytes and fibroblasts [28]. In some cases the cells resemble myofibroblasts, primitive mesenchymal cells, and cells having intermediate or mixed features. The presence of a homogeneous population of fibroblast-like cells has also been described [2]. Our case showed biphasic population with predominance of fibroblast-like spindle cells. Other histological features frequently described in BFH are the presence of multinucleated giant cells, abundant vascularity, and inflammatory infiltrate [2]. The main differential diagnosis of oral BFH includes nodular fasciitis, solitary fibrous tumour (SFT), neurofibroma (NF), and dermatofibroma (DF) [29]. CD34 positivity is a useful aid for distinction between BFH and SFT, the former reported as usually negative [8], although in our case the tumour showed weak immunoreactivity. Differentiation between BFH and NF can be based on S-100 positivity, more frequent mitoses and different fascicle configuration for the latter [29, 30]. BFH and DF show similar immunoreactivity [29, 31]. The negativity for SMA and S-100 could differentiate the lesion from leiomyosarcoma and neurogenic tumours [32].

 The treatment of choice is the complete resection of tumour, with an excellent prognosis and recurrence rate of almost zero [31, 34]. Fewer than 5% of cutaneous fibrous histiocytomas recur following local excision, and in the mouth most reported cases featured no recurrences [35]. BFH does not have metastatic potential [31, 34].

A review of the literature identified additional 6 cases of buccal mucosa BFH (Table 1). Most lesions were treated by a local excision without sacrificing structures that would cause major functional or cosmetic morbidity. One further paper reporting BFH of the oral mucosa was identified [36], however full-text article was not available to us for verification of the exact location of the tumours. 

## 4. Conclusion

We reported the clinical, microscopic, and immunohistochemical aspects of a case of BFH of the buccal mucosa. Although rare, BFH must be considered in the differential diagnosis of oral soft tissue tumors.

The prognosis of BFH is excellent. The results of this study support local excision as definitive treatment of BFH of buccal mucosa. When pathologically clear margins are found, the incidence of local recurrence is unlikely. Incomplete excision or enucleation may result in significant percentage of recurrence [37]. Radiation therapy and chemotherapy have currently no role in the management of BFH. 

A thorough knowledge of this lesion is important to the head and neck surgeon, who will provide the primary management of this rare lesion.

## Figures and Tables

**Table 1:** Buccal mucosa BFH: review of the literature.

Reference	Age (yr)	Sex	Treatment	FU length (months)	Outcome
O'Brien and Stout, 1964 [21]	50	F	LE	24	NED
Alonso del Hoyo et al., 1976 [33]	*	*	*	*	*
Hoffman and Martinez, 1981 [9]	8	M	LE	14	NED
Bielamowicz et al., 1995 [2]	25	M	LE	24	NED
Femiano et al., 2001 [4]	32	M	LE	**	**
Alves et al., 2003 [29]	26	F	LE	24	NED

LE: Local excision; FU: Follow-up; NED: No evidence of disease.

*Full-text article not available to authors. Case reported in the Hoffman and Martinez (1981) paper [9].

**Information not available in the original article.
